# Effects of a Velocity-Based Complex Training Program in Young Female Artistic Roller Skating Athletes

**DOI:** 10.5114/jhk/159654

**Published:** 2023-01-20

**Authors:** André Rebelo, João R. Pereira, Diogo V. Martinho, João Valente-dos-Santos

**Affiliations:** 1 CIDEFES, Centro de Investigação em Desporto, Educação Física e Exercício e Saúde, Universidade Lusófona, Lisboa, Portugal.; 2 COD, Center of Sports Optimization, Sporting Clube de Portugal, Lisbon, Portugal.; 3 Research Unity in Sport and Physical Activity (CIDAF, UID/DTP/04213/2020), Faculty of Sport Sciences and Physical Education, University of Coimbra, Coimbra, Portugal.; 4 Polytechnic of Coimbra, Coimbra Health School, Dietetics and Nutrition, Coimbra, Portugal.

**Keywords:** French Contrast Method, explosive strength, reactive strength index, load-velocity profile, figure skating, postactivation performance enhancement

## Abstract

Complex training consists of a near maximal strength effort followed by a biomechanically similar explosive exercise. One of many complex training methods that have been proposed is the French Contrast Method. The aim of this study was to analyze the effects of the French Contrast Method on maximal strength and power of young female artistic roller skating athletes with the help of velocity-based training to prescribe the intervention program. Eighteen female artistic roller skating athletes, divided into an experimental group (EG) and a control group (CG), participated in this study. The EG performed complex training via the French Contrast Method. The CG did not perform any additional training besides their regular roller skating practices. All participants were tested on the 1-RM back squat and hip thrust, the load-velocity profile assessment of both exercises previously stated, the countermovement jump, and the drop jump. A significant increase in mean concentric velocity (MCV) of the hip thrust exercise from 10 to 60% of 1-RM in the EG was observed. Significant differences between groups were observed for the MCV of the hip thrust from 10 to 90% of 1-RM. There were also significant increases in the 1-RM back squat and 1-RM hip thrust over time in the EG. For the vertical jump variables, there were significant differences between groups for both contact time and the reactive strength index with and without an arm swing. The results of this study suggest that a 6-week training intervention with the use of the French Contrast Method can significantly improve maximal strength and power.

## Introduction

Artistic roller skating consists of seven distinct disciplines: figures, freestyle, pairs, couple dance, solo dance, show, and precision. Athletes must perform different types of elements such as spins, jumps, lifts, and throws accordingly with their discipline (Rebelo et al., 2022b). The neuromuscular activity of the lower limb muscles is greater during jumps with a larger number of rotations in the air and the activity of some muscle groups, such as the gluteus maximus, vary depending on the type of the jump performed (Pantoja et al., 2014). To be able to do jumps with three rotations in the air, not only skaters need to rotate faster, but also jump higher (King, 2005). Therefore, striving to achieve the optimal jumping technique, by using different strength training modes for various leg muscles seems to be a crucial element of the artistic skaters’ training process.

In mechanical terms, power can be defined as the force applied multiplied by the velocity of movement (Knuttgen and Kraemer, 1987). The ability to produce high muscular power is considered one of the most important factors in different sports (Baker and Nance, 1999), including artistic roller skating (Rebelo et al., 2022a). Thus, optimizing either of these components (i.e., force or velocity) can lead to increased power production. For this reason, there are many studies analyzing the effects of different training methods on power, such as plyometric training (Markovic, 2007), resistance training (Channell and Barfield, 2008), or the combination of both (Fatouros et al., 2000).

An advanced training strategy for improving powerful performance (e.g., power), particularly in young and high-level athletes, is to combine maximal or near-maximal resistance exercises followed by plyometric or ballistic exercises (Prieske et al., 2020). This approach is utilized in what is known as complex training (Ebben, 2002) and has shown positive results on the power and agility development of young female athletes (Chaabene et al., 2021; Hammami et al., 2021). The improvements in maximal voluntary strength and power following complex training have been attributed to the postactivation performance enhancement (PAPE) (CuencaFernández et al., 2017).

One of many complex training methods that have been proposed to maximize the PAPE phenomenon is the French Contrast Method which consists of four consecutive exercises (Dietz and Peterson, 2012): 1. resistance exercise with the maximal load; 2. plyometric exercise; 3. resistance exercise maximizing power production; 4. accelerated or assisted short ground contact plyometric exercise. Training loads for exercises one and three are usually between 85–95% and 30– 40% of 1-repetition maximum (1-RM), respectively (Dietz and Peterson, 2012). This approach, using a percentage of 1-RM, is often referred to the “percentage-based” approach to calculate training intensities (Flanagan and Jovanović, 2014). However, this method becomes very problematic when we consider the day-to-day fluctuations in strength, which have been shown to be as large as 18% above and below the previously tested 1-RM (Flanagan and Jovanović, 2014). Therefore, alternative methods such as velocity-based training (VBT) have been developed to provide accurate and objective data to support the prescription of resistance training (Weakley et al., 2021). This training method can be implemented across various facets of resistance training programming and support the prescription of training loads, sets and the number of repetitions with the help of the velocity variable (Weakley et al., 2021). According to previous research, velocity-based resistance training seems to be an adequate method to improve physical performance in young athletes (González-Badillo et al., 2015).

Nevertheless, strength and conditioning coaches need to be aware of the factors that affect maturation and interact with different training stimuli. The types of measures of maturity generally match the biological system under consideration and the most common measure of biological maturation includes somatic age (Tanner, 1990). The age at the maximum rate of growth is the most used marker of somatic maturity and centered around peak height velocity (PHV) (i.e., the time when children grow the fastest during their adolescent growth spurt) (Lloyd and Oliver, 2012). In girls, the age of peak height velocity (APHV) occurs around the age of 11–12 (Tanner, 1990) and there appear to be certain periods in which young athletes are more sensitive to particular types of training (Ford et al., 2011). Thus, it is important to evaluate the APHV of young athletes so that practitioners can understand the physical consequences of the interaction of training and maturation.

To the authors’ knowledge, no study has evaluated the impact of the combination of complex training and VBT in young female athletes. Thus, given the lack of literature on the effects of the aforementioned topic, the aim of the present study was to analyze the effects of the French Contrast Method on maximal strength and power of young female artistic roller skating athletes with the help of VBT. The authors hypothesized that the combination of VBT with the French Contrast Method would result in an improvement of various lower-body strength variables, such as maximal strength and power, in young female athletes.

## Methods

### Experimental Approach to the Problem

The study was designed to assess the effects of complex training on maximal strength and power development of young female artistic roller skating athletes. Two groups, i.e., an experimental group (EG) and a control group (CG), were selected for this purpose. The EG performed complex training via the French Contrast Method, twice a week, for 6 weeks, along with their regular artistic roller skating training. The CG did not perform any additional training besides their regular artistic roller skating practices. All athletes were tested on the 1-RM barbell back squat, 1-RM barbell hip thrust, the load-velocity profile (LVP) assessment of both exercises previously stated, the countermovement jump (CMJ), and the drop-jump (DJ) from a 25-cm box (DJ25).

### Participants

Eighteen female artistic roller skating athletes participated in this study. All athletes had a minimum of 4 years' experience competing in their respective sport and a minimum of 2 years of resistance training experience. Participants were randomly assigned to the EG (n = 9: age, 14.0 ± 1.1 years; APHV, 12.4 ± 0.3 years; maturity offset, +1.6 ± 1.0 years; body mass, 50.5 ± 7.9 kg; body height, 156.7 ± 6.6 cm) or the CG (n = 9: age, 14.3 ± 1.4 years; APHV, 12.4 ± 0.3 years; maturity offset, +1.8 ± 1.2 years; body mass, 53.2 ± 6.2 kg; body height, 157.9 ± 5.4 cm). The biological age of all participants was estimated using the maturity offset method (Moore et al., 2015). Athletes, parents, and coaches were informed about the purpose of the study, and informed consent was obtained from all subjects and parents before the study started. All procedures were approved by the ethics committee of the Lusófona University of Humanities and Technologies, and were conducted in accordance with the declaration of Helsinki for human studies of the World Medical Association (World Medical Association, 2013).

### Testing Procedures

One week before and one week immediately after the intervention, all participants were submitted to testing sessions. These sessions were distributed into three days, with a 48-h interval in between.

### Session One: 1-Repetition Maximum of the Back Squat and the Hip Thrust

Prior to the 1-RM assessment, participants performed a warm-up consisting of four minutes of jogging, three minutes of dynamic stretching and mobility (e.g., world’s greatest stretch, ankle mobilizations, arm circles), and three minutes of glute and core activation exercises (e.g., hip abductions, side plank, bird-dog). Participants then commenced the 1-RM assessment and completed an initial set of 5–10 repetitions with the empty bar; followed by 5–6 repetitions at ≈50% estimated 1-RM. This was increased to ≈70% estimated 1-RM for 3–5 repetitions, and finally ≈90% estimated 1-RM for a single repetition. At this stage, the researcher dictated load increases, until 1-RM was achieved using correct technique, through a full range of motion, within three to five attempts. The barbell back squat was performed with the bar secured across the upper trapezius musculature. For the lift to be valid, during the eccentric phase, the hips should have descended lower than the knees (i.e., below parallel). For the hip thrust, the barbell was positioned around shoulder width apart and the bench placed under the shoulder blades (Contreras et al., 2011). From the previous position, the barbell was raised off the ground via a powerful contraction from the hip extensors, and not from the lumbopelvic region, until the torso was parallel with the ground and a hip neutral position was reached (Contreras et al., 2011). Two-minute passive rest was allowed between the warm-up and three to four minutes between 1-RM attempts. There were five minutes of rest between exercises and the total duration of the session, with the warm-up included, was 45 minutes.

### Session Two: Load-Velocity Profile of the Back Squat and the Hip Thrust

Following the warm-up and the technique for both the back squat and the hip thrust stated, all participants were assessed for the load-velocity (L-V) profile accordingly with previous recommendations (Weakley et al., 2021). Briefly, athletes completed three repetitions with 20%, 40%, and 60% of 1-RM and one repetition with 80% and 90% of 1-RM. For sets that involved three repetitions (i.e., loads 20–60%), the repetition with the fastest mean concentric velocity (MCV) was recorded. For sets that involved only one repetition (i.e., loads 80–90%), the MCV of that repetition was kept for analysis. The individualized L-V profiles were designed by plotting the MCV against the relative load and then applying a line of the best fit to the data (Microsoft Excel 2019, Microsoft, Redmond, Washington, USA). A linear regression equation was then calculated and used to modify the training load in the L-V profile experimental session. For both assessments the FLEX device was used (Kinetic, Canberra, Australia), which uses optic lasers to quantify displacement and, therefore, compute velocity. The FLEX device has already been shown to be accurate and reliable (Weakley et al., 2021).

### Session Three: Countermovement Jump and the Drop Jump

Each participant’s CMJ and DJ was calculated from flight time (Bosco et al., 2004) with a contact mat system (ChronojumpBoscoSystem®, Barcelona, Spain). Acceptable reliability has previously been reported for this contact mat (Pueo et al., 2020). In any type of the artistic skating jump, upward motion of the free limbs affects the forces applied during take-off and has the potential to increase the impulse generated during this phase (King, 2005). For reasons related with sport specificity, the authors decided to include both jumping tests with an arm swing as well as without an arm swing (i.e., hands akimbo). Peak power for CMJ tests was calculated through Sayers et al.’s equation (1999).

For the DJ test, athletes dropped from a box of 25-cm height, which was placed 5 cm behind the contact mat. Athletes were instructed to take-off with 2 feet, land with both feet on the mat, and jump as quickly and high as possible, minimizing the ground contact time. For both tests, the best of three trials was recorded with a minimum of 90 s rest interval between subsequent trials. Variables such as contact time (CT), measured in seconds, jumping height (JH), measured in centimetres, and reactive strength index (RSI), calculated as JH, in meters, divided by the CT, in seconds were analysed.

### Complex Training Protocol

After testing sessions, the EG completed 6 weeks (twice a week on nonconsecutive days) of complex training, following the French Contrast Method. All training sessions were monitored and supervised by at least one experienced researcher to ensure the correct form. The training program is described in detail in Table 1.

**Table 1:** Complex training protocol across the intervention phase.

Exercise	Week 1		Week 2		Week 3		Week 4		Week 5		Week 6	
	Sets x Reps	% 1-RM	Sets x Reps	% 1-RM	Sets x Reps	% 1-RM	Sets x Reps	% 1-RM	Sets x Reps	% 1-RM	Sets x Reps	% 1-RM
Day 1												
Back Squat	3 x ?	≈85	3 x ?	≈87.5	3 x ?	≈90	3 x ?	≈85	3 x ?	≈87.5	3 x ?	≈90
Drop Jump	3 x 4	N/A	3 x 5	N/A	3 x 6	N/A	4 x 4	N/A	4 x 5	N/A	4 x 6	N/A
Barbell CMJ	3 x 4	30	3 x 5	35	3 x 6	40	4 x 4	30	4 x 5	35	4 x 6	40
Band-Assisted Vertical Jump	3 x 4	N/A	3 x 5	N/A	3 x 6	N/A	4 x 4	N/A	4 x 5	N/A	4 x 6	N/A
Day 2												
Hip Thrust	3 x ?	≈85	3 x ?	≈87.5	3 x ?	≈90	3 x ?	≈85	3 x ?	≈87.5	3 x ?	≈90
Broad Jump Bound	3 x 4	N/A	3 x 5	N/A	3 x 6	N/A	4 x 4	N/A	4 x 5	N/A	4 x 6	N/A
Band-Resisted KB Swing	3 x 4	N/A	3 x 5	N/A	3 x 6	N/A	4 x 4	N/A	4 x 5	N/A	4 x 6	N/A
Accelerated Alternating Bound	3 x 4/side	N/A	3 x 5/side	N/A	3 x 6/side	N/A	4 x 4/side	N/A	4 x 5/side	N/A	4 x 6/side	N/A

* CMJ = countermovement jump; KB = kettlebell; Reps = repetitions; ? = number of repetitions according to athlete’s velocity prescription

Briefly, a standardized warm-up routine like the testing sessions explained earlier, was used. The rest period between each training set was three to four minutes, the time between repetitions within a set was approximately 2 s, and the rest interval between exercises was 20 s (e.g., back squat – DJ – barbell CMJ – band-assisted jump – rest of three to four minutes before the next set). All back squat and hip thrust repetitions were performed with a self-selected, controlled eccentric velocity and the concentric phase was performed with maximal effort immediately after the eccentric phase. Using the FLEX device, athletes were prescribed a velocity range, which varied by ±0.05 m•s^-1^, with the external load being altered to meet this targeted velocity, but always with the intend to move the heaviest load possible. A velocity loss (VL) threshold of 20% was used to guide set termination. During subsequent sets, if initial repetition velocity was greater than ±0.06 m•s^-1^ of targeted velocity, an additional 30-s recovery period was provided, and the external load was adjusted by 4–5% of 1-RM. This method enables the construction of a reliable and accurate training program (Weakley et al., 2021). In addition, all participants were verbally encouraged to perform each repetition with maximal effort.

### Statistical Analysis

Intraclass correlation coefficients (ICCs) were calculated to determine the reliability of all testing variables within sessions. Interpretation of these values was conducted using Portney and Watkins ranges (Portney and Watkins, 2009), whereby values > 0.75 indicate good reliability, values ranging from 0.5 to 0.75 imply moderate reliability and values < 0.5 suggest poor reliability. Variability in the data was assessed via the calculation of coefficients of variation (CoVs); this analysis of absolute reliability provides information regarding within-trial variability expressed as a percentage. Descriptive data are presented as means and standard deviations (SD). Independent samples *t*-tests were used to determine differences in baseline testing variables and the normal distribution of the data was confirmed by the Shapiro-Wilk’s test. Repeated measures analysis of variance (ANOVA) was used to determine the improvements in various tests between groups. When statistically significant differences existed in baseline values, one-way analysis of covariance (ANCOVA) was employed, using the baseline value as the covariate and change of the score as the dependent variable. Change scores were calculated by subtracting the baseline value from the posttest value. The goodness of fit of the L-V relationships was assessed through the Pearson’s multivariate coefficient of determination (R^2^). The differences in the L-V profile (i.e., slope of the load-velocity profile, y-intercept and MCV from 10% of 1-RM to 100% of 1-RM in 10% increments) were also assessed with the effect size (ES) (Cohen’s *d*) and its 95% confidence interval (Hopkins et al., 2009). For between-group effects, estimates of ES were calculated using standardized differences in mean values (Comprehensive Meta-Analysis, Biostat, Englewood, NJ, USA), whereas the independent-group ES was used for within-group effects as suggested by Morris and DeShon (2002). Effect sizes were interpreted via within-subject analyses as <0.3, 0.9, 1.6, 2.5, and >4.0 for trivial, small, moderate, large, very large, and extremely large effects, respectively (Hopkins et al., 2009). Regarding the between-subject analyses, ES was interpreted as <0.2, 0.6, 1.2, 2.0, and >4.0 for small, moderate, large, very large, and extremely large effects, respectively (Hopkins et al., 2009). All analyses were performed using Statistical Package for Social Science (V. 27.0, SPSS Inc., Chicago, IL, USA) and statistical significance was set at an alpha level of 0.05.

## Results

Results for the Shapiro-Wilk’s tests of normality are depicted in Table S1 and Table S2 of the Supplemental File. All within-session measures of reliability are reported in Table S3 of the Supplemental File. All variables demonstrated good within-session reliability, ranging from 0.89 to 0.99 for all variables. Of all jumping metrics, the RSI from a DJ25 with an arm swing demonstrated the greatest variability within trials (CoV = 6.34%). In the L-V testing session, the relative load at 90% of 1-RM for the back squat demonstrated the greatest variability (CoV = 7.31%).

There were differences in CT from the DJ without an arm swing (EG: 0.21 ± 0.03 s; CG: 0.24 ± 0.03 s; *t* = −2.4 [df = 16]; *p* = 0.03) and from the DJ with an arm swing (EG: 0.23 ± 0.03 s; CG: 0.26 ± 0.02 s; *t* = −2.4 [df = 16], *p* = 0.03) between the EG and CG at baseline. There were no differences between groups in any other testing variables at baseline. The independent samples *t*-tests used to determine differences in baseline values can be seen in Table S4 of the Supplemental File.

**Figure 1:**
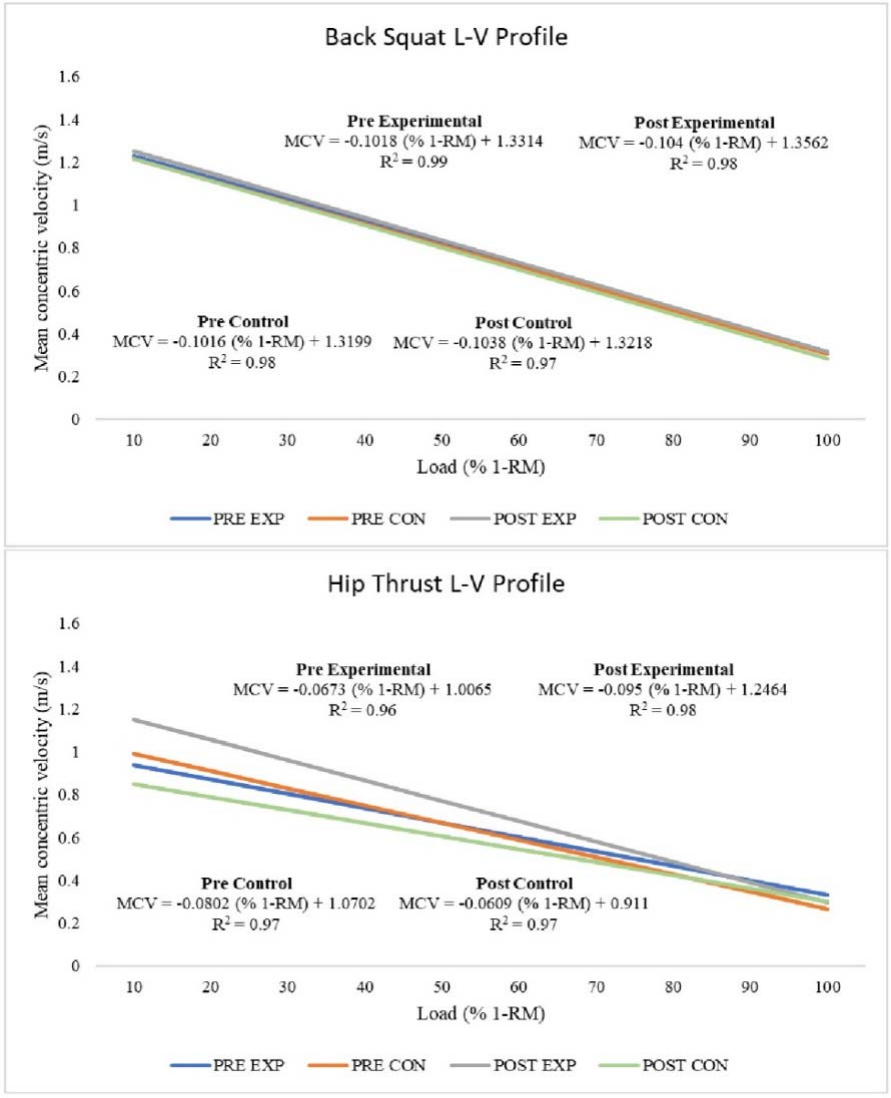
. Relationship between the relative load (% 1-RM) and mean concentric velocity (MCV) in the pre-intervention experimental group (blue line), pre-intervention control group (orange line), post-intervention experimental group (grey line), and post-intervention control group (green line), for the back squat (upper graphic) and the hip thrust (lower graphic). R^2^, Pearson’s multivariate coefficient of determination.

For the EG, the individualized L-V relationships showed a strong linearity for both the back squat (R^2^ = 0.99 [0.98–1.00]) and the hip-thrust (R^2^ = 0.96 [0.93–0.98]) during the pre-intervention period. Also, during the same period, the strength of the individualized L-V relationships was very strong for both the back squat (R^2^ = 0.99 [0.94–1.00]) and the hip-thrust (R^2^ = 0.98 [0.93–1.00]), for the CG. After the intervention phase, the strength of the individualized L-V relationships was very strong for both the back squat (R^2^ = 0.98 [0.92–1.00]; R2 = 0.97 [0.94–1.00]) and the hip-thrust (R^2^ = 0.99 [0.92–1.00]; R^2^ = 0.96 [0.92–1.00]), for the EG and for the CG, respectively. These relationships are depicted in Figure 1.

Results for MCV derived from L-V profiling are presented in Table 2. Significant group effects were found for MCV for the barbell hip thrust exercise at 10% 1-RM to 90% 1-RM with significant group by time interactions found at 10% 1-RM to 60% 1-RM, but with no significant time interactions found at any relative load. No significant time, group and group by time interactions were found for the MCV for the back squat exercise.

**Table 2:** Changes in the mean concentric velocity (m•s^-1^) attained at each relative load (%1-RM) from pre-intervention to post-intervention for the experimental group (EG) and the control group (CG) during the barbell back squat and the barbell hip thrust exercises.*†

Load (%1-RM)	EG (n = 9)			CG (n = 9)			ANOVA (*p*)			Between- group ES
	Pre	Post	ES	Pre	Post	ES	T	G	G × T	
**Back Squat**										
10 (m•s-1)	1.23 ± 0.07	1.25 ± 0.12	0.22	1.22 ± 0.13	1.22 ± 0.13	0.00	0.511	0.645	0.511	0.31
20 (m•s-1)	1.13 ± 0.07	1.15 ± 0.10	0.19	1.12 ± 0.11	1.11 ± 0.11	−0.04	0.679	0.594	0.477	0.33
30 (m•s-1)	1.03 ± 0.05	1.04 ± 0.09	0.24	1.02 ± 0.09	1.01 ± 0.09	−0.05	0.581	0.551	0.362	0.42
40 (m•s-1)	0.93 ± 0.04	0.94 ± 0.07	0.26	0.91 ± 0.07	0.91 ± 0.07	−0.10	0.669	0.451	0.293	0.48
50 (m•s-1)	0.82 ± 0.03	0.84 ± 0.06	0.30	0.81 ± 0.05	0.80 ± 0.06	−0.17	0.760	0.346	0.211	0.58
60 (m•s-1)	0.72 ± 0.03	0.73 ± 0.05	0.28	0.71 ± 0.04	0.70 ± 0.04	−0.29	0.948	0.208	0.185	0.64
70 (m•s-1)	0.62 ± 0.03	0.63 ± 0.04	0.26	0.61 ± 0.04	0.59 ± 0.04	−0.34	0.794	0.144	0.202	0.64
80 (m•s-1)	0.52 ± 0.04	0.52 ± 0.04	0.14	0.51 ± 0.05	0.49 ± 0.05	−0.38	0.571	0.231	0.265	0.57
90 (m•s-1)	0.42 ± 0.05	0.42 ± 0.04	0.13	0.41 ± 0.07	0.39 ± 0.07	−0.27	0.593	0.405	0.313	0.48
100 (m•s-1)	0.31 ± 0.07	0.32 ± 0.04	0.06	0.31 ± 0.08	0.28 ± 0.08	−0.25	0.523	0.546	0.382	0.41
**Hip Thrust**										
10 (m•s-1)	0.94 ± 0.13	1.15 ± 0.11	1.79	0.99 ± 0.14	0.85 ± 0.14	−1.01	0.480	0.004‡	0.002‡	1.69
20 (m•s-1)	0.87 ± 0.12	1.06 ± 0.09	1.75	0.91 ± 0.13	0.79 ± 0.12	−0.96	0.481	0.004‡	0.003‡	1.65
30 (m•s-1)	0.80 ± 0.10	0.96 ± 0.08	1.70	0.83 ± 0.12	0.73 ± 0.11	−0.88	0.460	0.003‡	0.004‡	1.59
40 (m•s-1)	0.74 ± 0.10	0.87 ± 0.07	1.57	0.75 ± 0.10	0.67 ± 0.09	−0.83	0.495	0.003‡	0.006‡	1.51
50 (m•s-1)	0.67 ± 0.08	0.77 ± 0.05	1.46	0.67 ± 0.09	0.61 ± 0.08	−0.74	0.513	0.003‡	0.010‡	1.42
60 (m•s-1)	0.60 ± 0.07	0.68 ± 0.05	1.23	0.59 ± 0.08	0.55 ± 0.07	−0.60	0.531	0.003‡	0.025‡	1.23
70 (m•s-1)	0.54 ± 0.06	0.58 ± 0.04	0.82	0.51 ± 0.07	0.49 ± 0.06	−0.36	0.635	0.005‡	0.127	0.83
80 (m•s-1)	0.47 ± 0.06	0.49 ± 0.05	0.35	0.43 ± 0.07	0.43 ± 0.05	−0.02	0.669	0.010‡	0.628	0.26
90 (m•s-1)	0.40 ± 0.05	0.39 ± 0.05	−0.17	0.35 ± 0.07	0.36 ± 0.04	0.31	0.817	0.029‡	0.489	0.36
100 (m•s-1)	0.33 ± 0.05	0.30 ± 0.07	−0.66	0.27 ± 0.07	0.30 ± 0.05	0.58	0.936	0.115	0.097	0.83

* ANOVA = analysis of variance; ES = effect size; Pre = pre-intervention/baseline testing; Post = post-intervention testing; T = time effect; G = group effect; G×T = group by time interaction; 1-RM = 1-repetition maximum. † Data presented as mean ± SD. ‡ Significant (<0.05).

The one-repetition maximum back squat improved after the 6-week intervention in the EG with small ES (*d* = 0.44) (Table 3). Similarly, the 1-RM hip thrust improved in the EG with a small ES (*d* = 0.61). Jumping variables, such as the CMJ (*d* = 0.003), CT without (*p* = 0.047) and with (*p* = 0.015) an arm swing, JH without an arm swing (*p* = 0.003), and RSI without (*p* < 0.001) and with (*p* = 0.023) an arm swing significantly improved in the EG over the 6-week intervention period. In the CG, CT without (*d* = 0.78) and with an arm swing (*d* = 0.97), decreased with a small and moderate ES, respectively. Significant group by time interactions were found for the 1-RM back squat (*p* < 0.001), 1-RM hip-thrust (*p* = 0.001), and RSI without (*p* < 0.001) and with (*p* = 0.006) an arm swing. Between-group ES showed large effects for the 1-RM back squat (*d* = 1.85), 1-RM hip thrust (*d* = 1.47), CT without (*d* = 1.29) and with (*d* = 1.28) an arm swing, and RSI with an arm swing (*d* = 1.40). Very large effects were observed for the RSI without an arm swing (*d* = 2.17). The countermovement jump without (*d* = 0.81) and with (*d* = 0.81) an arm swing and JH without (*d* = 0.84) and with (*d* = 0.86) an arm swing had a moderate ES between groups.

**Table 3:** Changes in the one-repetition maximum (1-RM) back squat and hip thrust, countermovement jump (CMJ) and drop jump variables from pre-intervention to post-intervention for the experimental group (EG) and the control group (CG).*†

Testing variable	EG (n = 9)				CG (n = 9)				ANOVA (*p*)			Between group ES
	Pre	Post	Δ (Post-Pre)	ES	Pre	Post	Δ (Post- Pre)	ES	T	G	G × T	
1-RM BS (kg)	70.56 ± 15.95	77.78 ± 17.11	7.22 ± 3.17	0.44	62.22 ± 16.93	60.28 ± 17.34	−1.94 ± 3.49	−0.11	0.004‡	0.122	<0.001‡	1.85
1-RM HT (kg)	132.22 ± 26.82	149.44 ± 29.20	17.22 ± 12.53	0.61	127.22 ± 28.41	126.67 ± 28.17	−0.56 ± 1.67	−0.02	0.001‡	0.305	0.001‡	1.47
CMJ (cm)	31.62 ± 4.65	33.06 ± 5.15	1.44 ± 1.04	0.29	28.84 ± 4.37	28.88 ± 4.69	0.03 ± 2.28	0.01	0.097	0.131	0.112	0.81
CMJ_as_(cm)	35.64 ± 5.24	36.53 ± 4.61	0.90 ± 1.21	0.18	32.71 ± 5.56	31.21 ± 6.05	−1.50 ± 3.93	−0.26	0.669	0.111	0.100	0.81
DJ25 CT (s)^a^	0.21 ± 0.03	0.19 ± 0.03	−0.01 ± 0.02	−0.48	0.24 ± 0.03	0.28 ±0.06	0.04 ± 0.06	0.78	-	0.030‡	-	1.29
DJ25 JH (cm)	26.19 ± 4.13	29.40 ± 4.63	3.21 ± 2.32	0.73	24.57 ± 4.62	25.23 ± 6.16	0.66 ± 3.51	−0.12	0.014‡	0.211	0.088	0.84
DJ25 RSI (m/s)	1.30 ± 0.33	1.56 ± 0.39	0.26 ± 0.13	0.72	1.04 ± 0.21	0.95 ± 0.32	−0.09± 0.17	−0.32	0.028‡	0.010‡	<0.001‡	2.17
DJ25_as_CT (s)^a^	0.23 ± 0.03	0.22 ± 0.03	−0.01 ± 0.03	−0.23	0.26 ± 0.02	0.30 ±0.06	0.04 ± 0.05	0.97	-	0.041‡	-	1.28
DJ25_as_JH (cm)	30.69 ± 6.49	34.13 ± 7.26	3.44 ±3.35	0.50	28.89 ± 7.57	28.72 ± 6.70	−0.17 ± 4.38	−0.02	0.094	0.274	0.067	0.86
DJ25_as_RSI (m/s)	1.36 ± 0.35	1.52 ± 0.27	0.16 ± 0.17	0.49	1.11 ±0.28	1.00 ±0.35	−0.12 ± 0.20	−0.37	0.665	0.017‡	0.006‡	1.40

* ANOVA = analysis of variance; Pre = pre-intervention/baseline testing; Post = post-intervention testing; ES = effect size; T = time effect; G = group effect; G×T = group by time interaction; BS = back squat; HT = hip thrust; CMJas = countermovement jump with an arm swing; DJ25 = drop jump from 25 cm; CT = contact time; JH = jump height; RSI = reactive strength index; DJ25as = drop jump from 25 cm with an arm swing. † Data presented as mean ± SD.‡ Significant (<0.05). ^a^ One-way ANCOVA using pre-test performance as a covariate

## Discussion

The present study aimed to assess the effects of a complex training program on maximal and explosive strength development of young female artistic roller skating athletes which had a minimum of 2 years of resistance training experience. The results of this study showed a significant increase in MCV of the hip thrust exercise from 10 to 60% of 1-RM in the EG. Significant differences between groups were observed for the MCV of the hip thrust from 10 to 90% of 1-RM. There were no significant differences in the MCV of the back squat exercise for both groups. There were significant increases in the 1-RM back squat and 1-RM hip thrust over time in the EG. For the vertical jump variables, there were significant increases in the JH and RSI from a DJ of 25-cm without an arm swing in the EG. No adverse effects were observed or reported during the intervention period.

Evidence has suggested that adolescents respond more favourably to muscle hypertrophy training than preadolescents due to the higher concentrations of certain hormones such as testosterone and growth hormone (Viru et al., 1999). Furthermore, research shows that adolescents respond better to training which targets both neural and structural development (e.g., strength training and plyometrics) (Viru et al., 1999). Since all subjects of this study had already experienced the PHV, it was somewhat expected that this sample of female athletes would improve their lower body maximal strength and vertical jumping ability.

Some studies have been conducted to examine the chronic effectiveness of the French Contrast Method. For example, vertical jump performance, measured with the CMJ without an arm swing, seems to improve after the implementation of this type of complex training in adult recreational athletes (Hernández-Preciado et al., 2018) and in female college athletes (Elbadry et al., 2019). Thus, the results of this study are in line with the current literature and confirm that the French Contrast Method is effective in improving vertical jumping ability in adolescent female athletes. Moreover, this study adds new evidence by showing that CT, JH, and RSI from a DJ of a 25-cm box significantly improved after a 6-week French Contrast Method intervention. Greater uptake of muscle slack and the buildup of high stimulation during the countermovement are the two main factors why the CMJ produces higher jumping heights (compared to jumps without countermovement) (Van Hooren and Zolotarjova, 2017). Although, the storage and utilization of elastic energy may also have a small contribution to the enhanced CMJ performance, this depends on several factors such as the amplitude of the countermovement and the capability of the individual to reduce muscle slack and quickly increase stimulation (Van Hooren and Zolotarjova, 2017).

Therefore, it can be hypothesized that the French Contrast Method is effective in increasing the uptake of muscle slack and the buildup of high stimulation during the countermovement. Another exercise used during the intervention program was the band-assisted jump. This exercise improves the rate of muscle shortening, which through a decrease in antagonist co-activation (Osternig et al., 1986) or an increase in cross-bridge cycling rates (Fitts, 2008), may promote an improvement in the jumping height ability. This is in line with the results of a study that showed that coaches can utilize assisted jumps to improve the jump height of male volleyball players (Sheppard et al., 2011).

This study used a novel approach by combining both contrast training methods with the VBT to prescribe the main exercises (i.e., the back squat and the hip thrust) loads and repetitions. There is a linear relationship between velocity and % 1-RM (Conceição et al., 2016), and with the accumulation of fatigue, exercise velocity decreases (González-Badillo et al., 2017). These concepts have been used to justify the application of velocity prescription of external loads and volumes irrespective of fluctuations in fatigue and athletes’ readiness. Results from the present study followed these tendencies and showed an almost perfect linear relationship between MCV and intensity (measured as % of 1-RM) in both the back squat and hip thrust exercises. This supports the use of MCV to prescribe the % 1-RM in the back squat and hip thrust exercises in young female artistic roller skating athletes. In fact, the goodness of fit of the individual L-V relationships obtained in the present study (R^2^ = 0.96 to 0.99) was similar to previously reported data for the squat exercise (Pérez-Castilla et al., 2020). This high linearity supports the use of the linear regression model. To the best of our knowledge, the present study addressed for the first time the full back squat and the hip thrust LVP in a sample of young female athletes. This is important since an individual L-V relationship should be used instead of generalized group equations for a more accurate prescription of the % 1-RM (Torrejón et al., 2019). According to the findings of the present study, the EG showed higher velocities after the intervention for the hip thrust at lower intensities (i.e., <70% 1-RM). However, no differences were observed in the velocities of the back squat exercise. This could be explained by the fact that this sample of athletes were already very proficient in the back squat technique, whereas their pre-intervention hip thrust velocities were lower than what is reported in the literature (de Hoyo et al., 2021). Therefore, it can be hypothesized that this group of young female athletes was not used to perform the barbell hip thrust with maximum concentric velocity in each repetition during the pre-testing period. Additionally, when individuals’ 1-RM is modified, previous investigations have confirmed that the relationship between movement velocity and the relative load remains the same (Davies et al., 2020). This study also showed that 6-week detraining from the usual resistance training regimen is not sufficient to induce statistically significant differences in the MCV for both the back squat and the hip thrust.

According to the results of the present study, the prescription of mean set velocities is effective in inducing changes in maximal strength of young female athletes. After extrapolating the regression equation, strength and conditioning coaches can create a velocity table in which each MCV corresponds to a percentage of 1-RM (Weakley et al., 2021). The training program carried out during this study used a fixed number of sets, however, the number of repetitions performed was flexible with the intention to diminish the differences between athletes’ physiological status. As maximal strength is considered a critical attribute for success in sports (Suchomel et al., 2016), including artistic roller skating (Rebelo et al., 2022a), this study indicates that the French Contrast Method using the VBT to prescribe training intensity is effective in improving the 1-RM of both the back squat and the hip thrust in young female athletes. In the back squat, since there were no differences in velocity in the EG after the intervention period, changes in muscular power after the application of the French Contrast Method were influenced by muscular strength development. This is in line with previous research that showed that changes in movement velocity did not dictate the variations in muscular power (de Vos et al., 2008). The results of the current study can help artistic roller skating athletes better cope with their sport demands. Increases in muscular strength achieved through physical training can modify subjects’ force-time characteristics which, in the end, will determine the magnitude of the impulse achieved during jumping tasks (Suchomel et al., 2016), like those observed in freestyle artistic roller skating athletes.

Although positive results were observed in most testing variables, it should be noted that all jumping tests were performed on a contact mat instead of a force plate. Even though contact mats are cheaper and easier to use than force plates (Glatthorn et al., 2011), researchers should be warned that the flight times predicted from contact mats are not always consistent when compared to the flight times predicted from force plates (Whitmer et al., 2015). Although the FLEX device provides an accurate alternative to more commonly used velocity measuring tools, the linear position transducers are still considered the best options when assessing barbell velocities (Weakley et al., 2020). Finally, the 6-week training duration for the current study is at the lower end of the minimum training duration threshold (i.e., 6–8 weeks) for neuromuscular adaptations (Folland and Williams, 2007). It is possible that results could have differed with longer duration.

Future research should evaluate the impact of the French Contrast Method in female adult athletes to understand what modifications can occur with older participants. Moreover, other complex training methods exist and it could be interesting to assess the physical impact of other training modalities in youth athletes. Lastly, in any type of the artistic skating jump, upward motion of the free limbs affects the forces applied during take-off. Considering that, future studies should analyze the impact of this type of training methodology on upper body strength and power.

Strength and conditioning coaches must be aware that athletes should always move the load with maximum intention, without sacrificing exercise technique, regardless of training loads used in order to enhance muscular power (Young and Bilby, 1993). As the hip and knee concentric extensions are present during the take-off of artistic roller skating jumps, coaches should be conscious of possible training interventions to strengthen these muscles in artistic roller skating athletes. Therefore, an off-rink strength training program that aims to improve the various zones of the lower body force-velocity curve should be implemented regularly.

## Conclusions

Maximizing athletic performance through strength and conditioning is the main goal of physical coaches. Consequently, coaches should implement an off-rink strength training program that aims to improve various zones of the lower body force-velocity curve to help artistic roller skating athletes better cope with the demands of this sport. Applying VBT is an effective way to prescribe training loads and repetitions and can be useful to enhance female athletes’ maximal strength and power. The results of this study suggest that a 6-week training intervention applying the French Contrast Method with VBT to prescribe the number of repetitions can improve the RSI, jump height, the 1-RM back squat and hip thrust.

## Figures and Tables

**Table S1a:** Shapiro-Wilk’s tests of normality of the pre-intervention measurements of the 1-RM and jumping tests.

Factor	Group	Shapiro-Wilk		
		Statistic	df.	Sig.
1-RM BS	EG CG	0.867 0.918	9 9	0.115 0.376
1-RM HT	EG CG	0.897 0.919	9 9	0.233 0.385
CMJ	EG CG	0.967 0.946	9 9	0.867 0.643
CMJas	EG CG	0.910 0.958	9 9	0.318 0.772
DJ25 CT	EG CG	0.914 0.894	9 9	0.342 0.221
DJ25 JH	EG CG	0.956 0.942	9 9	0.758 0.606
DJ25 RSI	EG CG	0.939 0.944	9 9	0.572 0.624
DJ25asCT	EG CG	0.974 0.916	9 9	0.927 0.364
DJ25asJH	EG CG	0.878 0.892	9 9	0.151 0.208
DJ25asRSI	EG CG	0.968 0.956	9 9	0.880 0.751

**Table S1b:** Shapiro-Wilk’s tests of normality of the pre-intervention measurements of the velocity assessments.

BS 10% 1-RM MCV	EG CG	0.932 0.953	9 9	0.500 0.718
BS 20% 1-RM MCV	EG CG	0.950 0.959	9 9	0.686 0.785
BS 30% 1-RM MCV	EG CG	0.959 0.966	9 9	0.793 0.857
BS 40% 1-RM MCV	EG CG	0.948 0.972	9 9	0.664 0.915
BS 50% 1-RM MCV	EG CG	0.964 0.975	9 9	0.835 0.932
BS 60% 1-RM MCV	EG CG	0.940 0.877	9 9	0.581 0.148
BS 70% 1-RM MCV	EG CG	0.936 0.916	9 9	0.543 0.360
BS 80% 1-RM MCV	EG CG	0.858 0.889	9 9	0.091 0.193
BS 90% 1-RM MCV	EG CG	0.841 0.848	9 9	0.059 0.071
BS 100% 1-RM MCV	EG CG	0.851 0.918	9 9	0.076 0.372
HT 10% 1-RM MCV	EG CG	0.939 0.918	9 9	0.572 0.378
HT 20% 1-RM MCV	EG CG	0.929 0.911	9 9	0.476 0.323
HT 30% 1-RM MCV	EG CG	0.940 0.894	9 9	0.582 0.218
HT 40% 1-RM MCV	EG CG	0.922 0.874	9 9	0.406 0.136
HT 50% 1-RM MCV	EG CG	0.908 0.894	9 9	0.301 0.220
HT 60% 1-RM MCV	EG CG	0.878 0.936	9 9	0.151 0.541
HT 70% 1-RM MCV	EG CG	0.851 0.948	9 9	0.055 0.668
HT 80% 1-RM MCV	EG CG	0.853 0.955	9 9	0.058 0.745
HT 90% 1-RM MCV	EG CG	0.852 0.947	9 9	0.078 0.658
HT 100% 1-RM MCV	EG CG	0.931 0.970	9 9	0.489 0.898

BS = back squat; HT = hip thrust; CMJ = countermovement jump; CMJas = countermovement jump with an arm swing; DJ25 = drop jump from 25 cm; CT = contact time; JH = jump height; RSI = reactive strength index;

DJ25as = drop jump from 25 cm with an arm swing; MCV = mean concentric velocity; 1-RM = one-repetition maximum; EG = experimental group; CG = control group.

**Table S2a:** Shapiro-Wilk’s tests of normality of the post-intervention measurements of the 1-RM and jumping tests.

Factor	Group	Statistic	df.	Sig.
1-RM BS	EG CG	0.879 0.906	9 9	0.152 0.291
1-RM HT	EG CG	0.859 0.934	9 9	0.093 0.525
CMJ	EG CG	0.979 0.914	9 9	0.957 0.343
CMJas	EG CG	0.956 0.894	9 9	0.754 0.221
DJ25 CT	EG CG	0.916 0.944	9 9	0.383 0.624
DJ25 JH	EG CG	0.902 0.933	9 9	0.263 0.511
DJ25 RSI	EG CG	0.975 0.974	9 9	0.935 0.929
DJ25asCT	EG CG	0.950 0.981	9 9	0.694 0.969
DJ25asJH	EG CG	0.989 0.897	9 9	0.995 0.233
DJ25asRSI	EG CG	0.956 0.923	9 9	0.752 0.415

**Table S2a:** Shapiro-Wilk’s tests of normality of the post-intervention measurements of the velocity assessments.

BS 10% 1-RM MCV	EG CG	0.929 0.950	9 9	0.468 0.691
BS 20% 1-RM MCV	EG CG	0.915 0.954	9 9	0.354 0.732
BS 30% 1-RM MCV	EG CG	0.891 0.953	9 9	0.203 0.719
BS 40% 1-RM MCV	EG CG	0.889 0.954	9 9	0.196 0.730
BS 50% 1-RM MCV	EG CG	0.887 0.929	9 9	0.185 0.476
BS 60% 1-RM MCV	EG CG	0.930 0.865	9 9	0.485 0.059
BS 70% 1-RM MCV	EG CG	0.908 0.856	9 9	0.302 0.086
BS 80% 1-RM MCV	EG CG	0.845 0.897	9 9	0.066 0.236
BS 90% 1-RM MCV	EG CG	0.895 0.883	9 9	0.223 0.167
BS 100% 1-RM MCV	EG CG	0.969 0.934	9 9	0.890 0.524
HT 10% 1-RM MCV	EG CG	0.964 0.927	9 9	0.842 0.455
HT 20% 1-RM MCV	EG CG	0.948 0.913	9 9	0.663 0.340
HT 30% 1-RM MCV	EG CG	0.918 0.912	9 9	0.376 0.331
HT 40% 1-RM MCV	EG CG	0.890 0.897	9 9	0.198 0.236
HT 50% 1-RM MCV	EG CG	0.935 0.867	9 9	0.526 0.115
HT 60% 1-RM MCV	EG CG	0.936 0.845	9 9	0.540 0.059
HT 70% 1-RM MCV	EG CG	0.898 0.834	9 9	0.243 0.056
HT 80% 1-RM MCV	EG CG	0.956 0.844	9 9	0.757 0.053
HT 90% 1-RM MCV	EG CG	0.982 0.877	9 9	0.975 0.145
HT 100% 1-RM MCV	EG CG	0.990 0.933	9 9	0.996 0.512

BS = back squat; HT = hip thrust; CMJ = countermovement jump; CMJas = countermovement jump with an arm swing; DJ25 = drop jump from 25 cm; CT = contact time; JH = jump height; RSI = reactive strength index; DJ25as =

drop jump from 25 cm with an arm swing; MCV = mean concentric velocity; 1-RM = one-repetition maximum; EG = experimental group; CG = control group.

**Table S3:** Within-session reliability (intraclass correlation coefficients [ICC] and coefficients of variation [CoV]) obtained from all trials of all testing variables.

Testing variable	ICC (95% CI)	CoV (%)
CMJ	0.99 (0.98-1.00)	1.89
CMJ_as_	0.99 (0.97-1.00)	2.04
DJ25 JH	0.97 (0.93-0.99)	4.07
DJ25 CT	0.92 (0.78-0.97)	5.75
DJ25 RSI	0.97 (0.92-0.99)	6.15
DJ25_as_JH	0.98 (0.95-0.99)	3.34
DJ25_as_CT	0.89 (0.70-0.96)	5.51
DJ25_as_RSI	0.97 (0.91-0.99)	6.34
BS 20% 1-RM MCV	0.96 (0.91-0.98)	2.28
BS 40% 1-RM MCV	0.96 (0.90-0.98)	2.43
BS 60% 1-RM MCV	0.95 (0.89-0.97)	2.36
BS 80% 1-RM MCV	0.86 (0.72-0.94)	5.91
BS 90% 1-RM MCV	0.81 (0.61-0.91)	7.31
HT 20% 1-RM MCV	0.94 (0.89-0.96)	2.45
HT 40% 1-RM MCV	0.95 (0.90-0.97)	2.34
HT 60% 1-RM MCV	0.89 (0.89-0.97)	3.54
HT 80% 1-RM MCV	0.87 (0.73-0.95)	5.06
HT 90% 1-RM MCV	0.83 (0.63-0.93)	6.94

CI = confidence interval; BS = back squat; HT = hip thrust; CMJ = countermovement jump; CMJ_as_ = countermovement jump with an arm swing; DJ25 = drop jump from 25 cm; CT = contact time; JH = jump height; RSI = reactive strength index; DJ25_as_ = drop jump from 25 cm with an arm swing; MCV = mean concentric velocity; 1-RM = one-repetition maximum.

**Table S4:** Differences in baseline testing variables using independent samples *t*-tests.

Testing variable	Independent sample *t*-test		
	*t*	df.	Sig.
1-RM BS	1.075	16	0.298
1-RM HT	0.384	16	0.706
CMJ	1.307	16	0.210
CMJas	1.149	16	0.267
DJ25 JH	0.783	16	0.445
DJ25 CT	-2.427	16	0.027†
DJ25 RSI	1.968	16	0.067
DJ25_as_JH	0.540	16	0.596
DJ25_as_CT	-2.384	16	0.030†
DJ25_as_RSI	1.643	16	0.120
BS 20% 1-RM MCV	0.316	16	0.756
BS 40% 1-RM MCV	0.425	16	0.677
BS 60% 1-RM MCV	0.693	16	0.498
BS 80% 1-RM MCV	0.544	16	0.594
BS 90% 1-RM MCV	0.322	16	0.752
HT 20% 1-RM MCV	-0.652	16	0.524
HT 40% 1-RM MCV	-0.259	16	0.799
HT 60% 1-RM MCV	0.337	16	0.741
HT 80% 1-RM MCV	1.411	16	0.178
HT 90% 1-RM MCV	1.951	16	0.069

BS = back squat; HT = hip thrust; CMJ = countermovement jump; CMJ_as_ = countermovement jump with an arm swing; DJ25 = drop jump from 25 cm; CT = contact time; JH = jump height; RSI = reactive strength index; DJ25_as_ = drop jump from 25 cm with an arm swing; MCV = mean concentric velocity; 1-RM = one-repetition maximum. † Significant.

## SUPPLEMENTAL MATERIAL CONTENTS

Table S1. Shapiro-Wilk’s tests of normality of the pre-intervention measurements.

Table S2. Shapiro-Wilk’s tests of normality of the post-intervention measurements.

Table S3. Within-session reliability (intraclass correlation coefficients [ICC] and coefficients of variation [CoV]) obtained from all trials of all testing variables.

Table S4. Differences in baseline testing variables using independent samples *t*-tests
